# Prenatal Exposure to Lipopolysaccharide Alters Renal DNA Methyltransferase Expression in Rat Offspring

**DOI:** 10.1371/journal.pone.0169206

**Published:** 2017-01-19

**Authors:** Jing Wang, Jinghong Cui, Rui Chen, Youcai Deng, Xi Liao, Yanling Wei, Xiaohui Li, Min Su, Jianhua Yu, Ping Yi

**Affiliations:** 1 Department of Obstetrics and Gynecology, Research Institute of Surgery, Daping Hospital, Third Military Medical University, Chongqing, China; 2 Institute of Materia Medica, College of Pharmacy, Third Military Medical University, Chongqing, China; 3 Department of Gastroenterology, Research Institute of Surgery, Daping Hospital, Third Military Medical University, Chongqing, China; 4 The Ohio State University Comprehensive Cancer Center, Columbus, Ohio, United States of America; University of Southampton, UNITED KINGDOM

## Abstract

Prenatal exposure to inflammation results in hypertension during adulthood but the mechanisms are not well understood. Maternal exposure to lipopolysaccharide (LPS) alters interleukin-6 (IL-6) and tumor necrosis factor-α (TNF-α) levels in the fetal environment. As reported in many recent studies, IL-6 regulates DNA methyltransferases (DNMTs) through the transcription factor friend leukemia virus integration 1 (Fli-1). The present study explores the role of intrarenal DNMTs during development of hypertension induced by prenatal exposure to LPS. Pregnant rats were randomly divided into four treatment groups: control, LPS, pyrrolidine dithiocarbamate (PDTC, a NF-κB inhibitor), and the combination of LPS and PDTC. Expression of IL-6, Fli-1, TNF-α, DNMT1 and DNMT3B was significantly increased in the offspring of LPS-treated rats. Global DNA methylation level of renal cortex also increased dramatically in rat offspring of the LPS group. Prenatal PDTC administration reversed the increases in gene expression and global DNA methylation level. These findings suggest that prenatal exposure to LPS may result in changes of intrarenal DNMTs through the IL-6/Fli-1 pathway and TNF-α, which probably involves hypertension in offspring due to maternal exposure to inflammation.

## Introduction

The developmental origins of health and disease (DOHaD) approach has evolved over the past 20 years [1]. It is known that intrauterine life can affect the incidence of late onset of diseases such as hypertension [2, 3].

Epigenetic modifications of key genes have been proposed as probable mechanisms in the developmental programming of cardiovascular and metabolic diseases in offspring because of maternal exposure to adverse environments [4]. Bogdarina et al. showed that the proximal promoter of the AT1b gene is significantly undermethylated and expression of the AT1b angiotensin receptor gene is upregulated in the adrenal gland during the development of prenatal limited food intake-induced hypertension [5]. This observation indicates that an adverse environment during early life can alter the expression of the AT1b gene via methylation [5].

Our previous studies have shown that maternal exposure to lipopolysaccharide (LPS) and zymosan results in hypertension and higher inflammatory responses in rat offspring [2, 3]. Pyrrolidine dithiocarbamate (PDTC), a nuclear factor (NF)-κB inhibitor, prevents hypertension in these offspring [2, 3]. However, the pathogenesis has not yet been reported, to our knowledge. Some research suggest that IL-6 and TNF-α expression could be upregulated via the NF-κB pathway with LPS treatment [6, 7]. Urakubo et al. found that maternal exposure to LPS alters the levels of proinflammatory cytokines including interleukin-6 (IL-6) and tumor necrosis factor-α (TNF-α) in the fetal environment [8]. Inflammatory cytokines can induce alterations in the expression and activity of DNA methyltransferases (DNMTs; cytosine-5-methyltransferases) in some cancer cells [9–12]. IL-6 upregulates DNMTs through the transcription factor friend leukemia virus integration 1 (Fli-1) [11] and TNF-α can stimulate DNMT3B by upregulation of NF-κB [12].

Based on these findings, we mainly elucidate that maternal inflammatory stimuli convert the expression levels of DMNTs and induce epigenetic modifications by upregulating expression of inflammatory cytokines in offspring, leading to adult hypertension. In the present study, we established the hypertensive rat model induced by prenatal exposure to LPS to detect expression levels of IL-6, Fli-1, TNF-α, DNMT1, and DNMT3 in the renal cortex tissue of the offspring and investigated whether DNA methylation is associated with developmental programming of hypertension.

## Materials and Methods

### Animals

Nulliparous pregnant Sprague-Dawley rats were purchased from the Animal Centre of the Third Military Medical University (Chongqing, China). The staff made vaginal examination of the females at 7:00 on the next day after mating and females had a vaginal plug that was defined as gestational day 0. All animals had free access to standard laboratory rat chow and water. They were housed individually throughout pregnancy at a constant temperature (24°C) under a 12-h light-dark cycle until childbirth.

This study was performed in strict accordance with the recommendations in the Guide for the Care and Use of Laboratory Animals of the National Institutes of Health. The protocol was approved by the local animal ethics committee at the Third Military Medical University. All surgery was performed under urethane anesthesia, and every effort was made to minimize suffering.

### Dams and litters

Pregnant rats were randomly divided into four groups (n = 4 in each): control, LPS, PDTC, and LPS+PDTC. The rats in these groups were intraperitoneally administered 0.5 ml normal saline, 0.79 mg/kg LPS (Sigma, St Louis, MO, USA), 100 mg/kg PDTC (Sigma), or LPS plus PDTC, respectively. LPS was administered on gestational days 8, 10, and 12, whereas PDTC was administered daily from day 8 to 14 during gestation. Rats in the LPS group were administered normal saline on gestational days 9, 11, 13, and 14 and rats in the control group were administered normal saline every day from day 8 to 14.

In each group, pups were raised by a lactating mother until 4 weeks of age and then were separated into new cages with four to five rats per cage according to gender. Sixteen pups (eight males and eight females) were randomly selected from each group for physiological measurements, and then the 6 pups (three males and three females) for molecular biological experiments at 6 weeks or 12 weeks of age were randomly selected from the rest animals. The excess animals were culled.

### Body weight measurement

Rat offspring were weighed by an electronic balance every 2 weeks from 4 to 12 weeks of age.

### Blood pressure measurement

Systolic blood pressure (SBP) was measured in conscious rat offspring in each group at 6, 8, 10, and 12 weeks of age using the tail-cuff method (ML 125, Powerlab, AD Instruments, Castle Hill, Australia), as described previously [2]. Before the measurement, the rats were placed inside a warming chamber (about 34°C) for 15 min. Then, the rats were placed in plastic restraints. A cuff with a pneumatic pulse sensor was attached to the tail, setting to the proximal end of the tail. In each rat, the mean SBP was calculated from at least three SBP recordings. The rats were allowed to habituate to this procedure for 7 days before the experiments.

### Collection of kidney tissue

Offspring rats at 6 and 12 weeks of age were anesthetized with urethane (20%). After decapitation, the kidney tissue was abscised and stored at -80°C. The renal cortex tissue was separated precisely during the molecular experiment.

### Real-time RT-PCR

The mRNA expression of IL-6, Fli-1, DNMT1, DNMT2, and DNMT3 in renal cortex tissue was assessed by real-time, RT-PCR, when the offspring were at 6 and 12 weeks of age according to a previously described method [13]. A RNA simple Total RNA Kit (TIANGEN Biotech, Beijing, China) was used to extract total RNA from kidneys. Total RNA (1 μg) was then reverse transcribed into cDNA using a PrimeScript^™^ RT Reagent Kit with gDNA Eraser (TaKaRa Biotechnology, Dalian, China). β-Actin served as the internal control. The PCR primers were designed by Premier 5.0 (Premier Biosoft international, Palo Alto, CA, USA), based on the published nucleotide sequences. Each real-time PCR was carried out in a total volume of 25 μl with SYBR Premix Ex Taq II (Tli RNaseH Plus) (TaKaRa Biotechnology) under the following conditions: 30 s at 95°C and then 40 cycles at 95°C for 15 s, 60°C for 15 s, and 72°C for 20 s. PCRs were performed using an Eppendorf Mastercycler ep realplex system (Eppendorf, Hamburg, Germany). The cycle threshold (Ct) values were normalized to the expression levels of β-actin. The equation 2^-ΔΔCt^ was used to calculate the relative expression ratio of each mRNA. The primers for each gene were as follows: IL-6 (forward: 5′-CTT CCA GCC AGT TGC CTT CTT G-3′; reverse: 5′-GTC TGT TGT GGG TGG TAT CCT C-3′); Fli-1 (forward: 5′-TAT GGC TTG ATG GAG ATT GAC ACT-3′; reverse: 5′-CCT GAG GTA ACT GAG GTG CGA C-3′); DNMT1 (forward: 5′-CGT CAT AAC CAA TAA ACT TCG CT-3′; reverse: 5′-TTG TCT GGA AGC AGG GTC G-3′); DNMT3A (forward: 5′-TGT GAA TGA TAA GCT GGA GTT GC-3′; reverse: 5′-GGT GGT AAT GGT CCT CAC TTT G-3′); DNMT3B (forward: 5′-GTG CGT CGT TCA GGC AGT AG-3′; reverse: 5′-AAG CGG CAA AGT CAA TGG TT-3′); β-actin (forward: 5′- ACG GTC AGG TCA TCA CTA TCG-3′; reverse: 5′-GGC ATA GAG GTC TTT ACG GAT G-3′).

### Enzyme-linked immunosorbent assay (ELISAs)

IL-6 and TNF-α protein levels were measured using a Rat IL-6 ELISA Kit (CUSABIO Life Science Inc., Wuhan, China) and Rat TNF-α ELISA Kit (CUSABIO Life Science Inc.), respectively, according to the manufacturer’s protocols. 100 mg renal cortex tissue was rinsed with 1×PBS, homogenized in 1 ml of 1×PBS and stored overnight at -20°C. After two freeze-thaw cycles to break the cell membranes, the homogenates were centrifuged for 5 min at 5000 g, 4°C. The supernate was removed and assayed immediately.

### Western blot analysis

Western blotting was performed as described previously [14]. Proteins in renal cortex tissue of 6- and 12-week-old rats were extracted. 50 mg renal cortex tissue was homogenized in 1 ml mixed lysis buffer containing the T-PER^™^ tissue protein extraction reagent (Pierce Chemical, Rockford, IL, USA) and the protease inhibitor cocktail (Sigma). Homogenates were centrifuged at 4°C for 10 min at 10000 g and supernatants were collected. Protein concentration was measured using a Bicinchoninic acid kit (Beyotime Biotechnology, Shanghai, China). After denaturation and electrophoresis on SDS-polyacrylamide gels, the separated proteins were transferred to nitrocellulose membranes. The membranes were then blocked for 1 h at room temperature in 5% dry milk/Tris-buffered saline (TBS; 20 mM Tris-HCl, pH 7.5, and 0.15 M NaCl) containing 0.1% Tween-20.

After incubation with primary antibodies [anti-Fli-1 (1:1500, SC-356, Santa Cruz Biotechnology, Santa Cruz, CA, USA), anti-DNMT 1 (1:500, SC-20701, Santa Cruz Biotechnology), anti-DNMT3A (1:500, SC-20703, Santa Cruz Biotechnology), anti-DNMT3B (1:500, SC-20704, Santa Cruz Biotechnology), or anti-GAPDH (1:5000, 2118S, Cell Signaling Technology, Beverly, MA, USA)] in TBS at 4°C overnight, the membranes were incubated with a peroxidase-conjugated secondary antibody in TBS at room temperature for 1 h. Specific bands were detected by enhanced chemiluminescence and recorded on X-ray film. Quantity One software (Bio-Rad, Hercules, CA, USA) was used to quantify the band intensities. Data were normalized to GAPDH levels.

### Global methylation status

Global methylation levels for renal cortex tissue were determined by the MethylFlash^™^ Methylated DNA Quantification Kit (Epigentek, Brooklyn, NY, USA), according to the manufacturer’s protocols with 100 ng of genomic DNA.

### Statistical analysis

One-way analysis of variance (ANOVA) was used for statistical analysis. All results are presented as the means ± standard deviation (SD). A P-value of less than 0.05 was considered statistically significant. For RT-PCR and western blot experiments, data were first normalized to the internal controls. All analyses were performed with SPSS 18.0 (SPSS Inc., Chicago, IL, USA).

## Results

### Assessment of body weights and SBP of rat offspring

**Fig 1:**
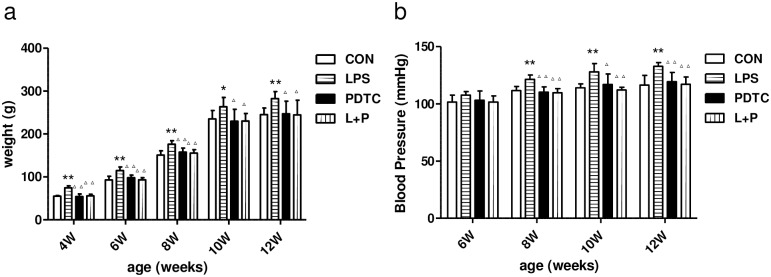
Effects of prenatal exposure to LPS on body weight (a) and SBP (b) in rat offspring. Data are presented as the means ± SD (n = 16 in each group; eight females and eight males). *P<0.05 and **P<0.01 compared with controls; ^Δ^P<0.05 and ^ΔΔ^P<0.01 compared with offspring of the LPS group. CON, Control; LPS, lipopolysaccharide; PDTC, pyrrolidine dithiocarbamate; L+P, LPS+PDTC.

Body weights of rat offspring at 4–12 weeks of age were measured every 2 weeks. The body weights of rat offspring in the LPS group was significantly higher than those in the control group from 4 weeks of age (P<0.05) and remained at the higher level at 12 weeks of age. However, the body weights of rat offspring in LPS+PDTC were similar to the control groups (Fig 1a). As shown in Fig 1b, the mean SBP of offspring in the LPS group was significantly higher than those in the control group, which was not observed in the LPS+PDTC group from 8 to 12 weeks of age.

### IL-6, Fli-1 and DNMT mRNA levels in the kidney

**Fig 2:**
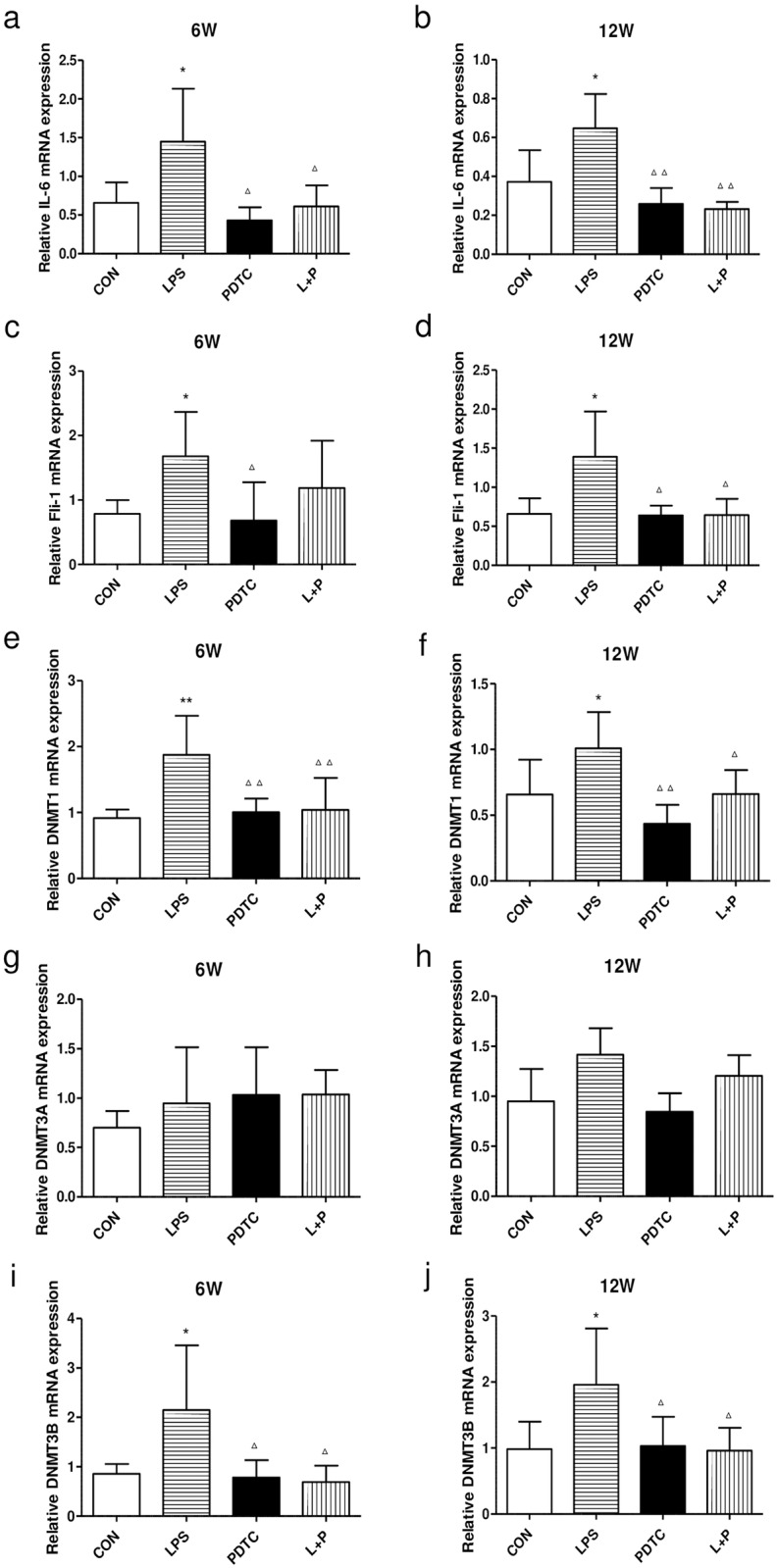
Effects of prenatal exposure to LPS on the mRNA expression of IL-6 (a, b), Fli-1 (c, d), DNMT1 (e, f), DNMT3A (g, h), and DNMT3B (i, j) in the renal cortex of rat offspring at 6 and 12 weeks of age. Data are presented as the means ± SD (n = 6 in each group; three females and three males). *P<0.05 and **P<0.01 compared with controls; ^Δ^P<0.05 and ^ΔΔ^P<0.01 compared with offspring of the LPS group. CON, Control; LPS, lipopolysaccharide; PDTC, pyrrolidine dithiocarbamate; L+P, LPS+PDTC.

To obtain precise results, we examined the expression of IL-6, Fli-1, and DNMT in each sample by Real-time RT-PCR. Compared with the control group, mRNA expression of IL-6, Fli-1, DNMT1 and DNMT3B in the renal cortex was increased dramatically in rat offspring of the LPS group and reversed in the LPS+PDTC group at both 6 and 12 weeks of age, except for mRNA expression of Fli-1 at 6 weeks of age (Fig 2a–2f, 2i and 2j). There was no significant difference in the expression of DNMT3A among these groups (Fig 2g and 2h).

### ELISA analysis of IL-6 and TNFα in the kidney

**Fig 3:**
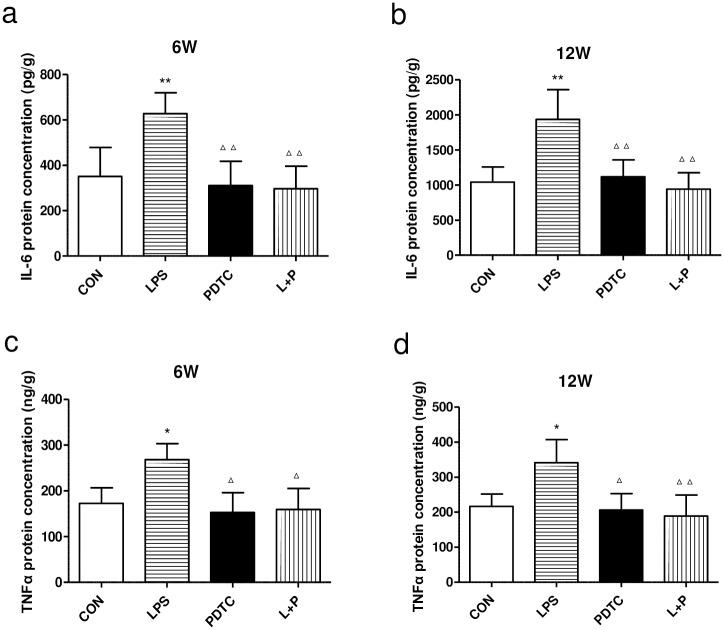
Effects of prenatal exposure to LPS on the protein levels of IL-6 (a, b) and TNFα (c, d) in the renal cortex of rat offspring as determined by ELISAs. Data are presented as the means ± SD (n = 6 in each group; three females and three males). *P<0.05 and **P<0.01 compared with controls; ^Δ^P<0.05 and ^ΔΔ^P<0.01 compared with offspring of the LPS group. CON, Control; LPS, lipopolysaccharide; PDTC, pyrrolidine dithiocarbamate; L+P, LPS+PDTC.

Compared to the control, IL-6 and TNFα protein levels in the renal cortex showed significant increases in offspring of the LPS group at both 6 and 12 weeks of age, whereas the PDTC treatment inhibited these increases (Fig 3).

### Western blot analysis of Fli-1 and DNMTs in the kidney

**Fig 4:**
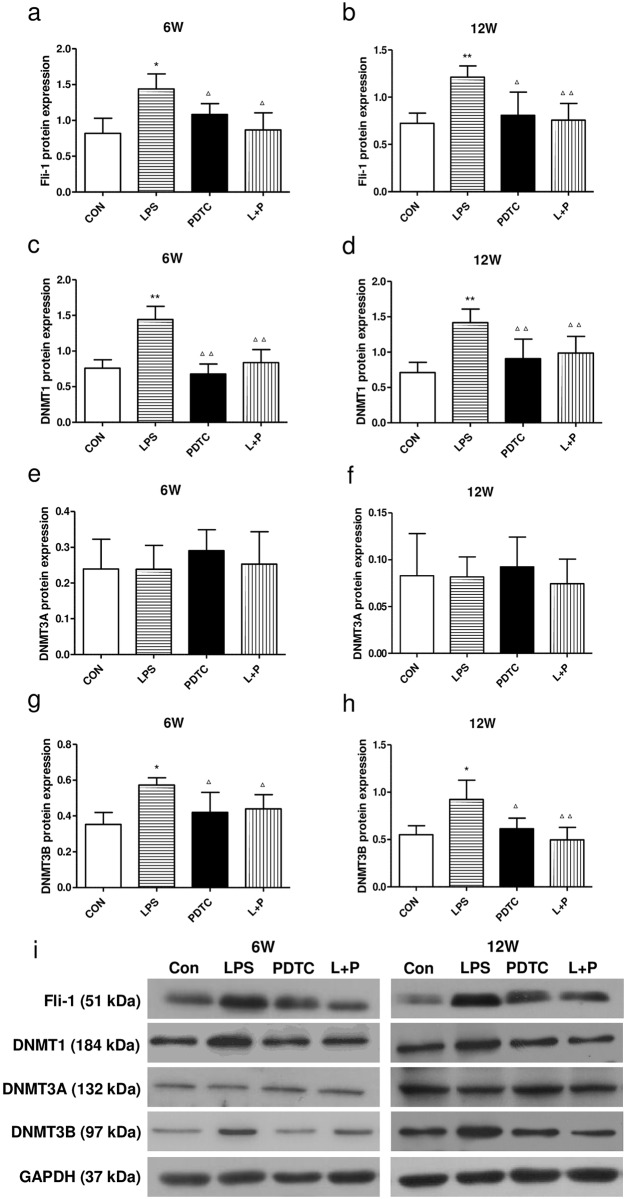
Effects of prenatal exposure to LPS on the protein expression of Fli-1 (a, b), DNMT1 (c, d), DNMT3A (e, f), and DNMT3B (g, h) in the renal cortex of rat offspring as measured by western blotting (i) at 6 and 12 weeks of age. Data are presented as the means ± SD (n = 6 in each group; three females and three males). *P<0.05 and **P<0.01 compared with controls; ^Δ^P<0.05 and ^ΔΔ^P<0.01 compared with offspring of the LPS group. CON, Control; LPS, lipopolysaccharide; PDTC, pyrrolidine dithiocarbamate; L+P, LPS+PDTC.

To further validate these findings, western blot analysis was used to examine Fli-1 and DNMTs proteins in the renal cortex. Compared with the control group, the protein expression of Fli-1, DNMT1 and DNMT3B in the renal cortex was increased significantly in rat offspring of the LPS group and reversed in the LPS+PDTC group at both 6 and 12 weeks of age (Fig 4a–4d and 4g–4i). However, there was no significant differences in the expression of DNMT3A among these groups (Fig 4e, 4f and 4i).

### Global methylation levels in the kidney

**Fig 5:**
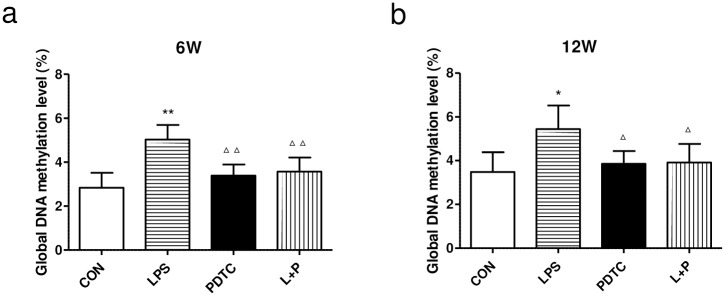
Effects of prenatal exposure to LPS on the global DNA methylation level in the renal cortex of rat offspring at 6 (a) and 12 (b) weeks of age. Data are presented as the means ± SD (n = 6 in each group; three females and three males). *P<0.05 and **P<0.01 compared with controls; ^Δ^P<0.05 and ^ΔΔ^P<0.01 compared with offspring of the LPS group. CON, Control; LPS, lipopolysaccharide; PDTC, pyrrolidine dithiocarbamate; L+P, LPS+PDTC.

To investigate whether DNA methylation is associated with developmental programming of hypertension, global methylation levels in renal cortex tissue were measured. Compared to the control group, global DNA methylation levels of renal cortex tissue was increased dramatically in rat offspring of the LPS group and reversed in the LPS+PDTC group at both 6 and 12 weeks of age (Fig 5a and 5b).

## Discussion

Non-communicable diseases, which are the main causes of death worldwide, constitute almost two-thirds of all global deaths. Once exposed to a nutritional imbalance or environmental contaminants in the uterus and early life time, it may affect normal adult physiological functions such as metabolism, immune functions, and reproductive functions, which results in the risk of adult diseases [1]. The potential mechanisms are likely based on activation of certain common molecular pathways, leading to changes in the expression of specific genes and induction of specific phenotypes but without DNA sequence modification. Evidence suggests that developmental exposure to a nutritional imbalance or environmental contaminants can not only directly activate the receptors that stimulate gene expression but also activate or inhibit enzymes and pathways which are responsible for DNA methylation, histone modifications, and non-coding RNA expression [1, 4, 15].

In fact, some epigenetic marks including DNA methylation and histone modifications generally undergo substantial changes which can affect the expression of genes which are essential for both early life development and physiological functions in later life. At the same time, epigenetic modifications are often stable from one cell generation to the next and can be transmitted transgenerationally in some cases [1]. Epigenetic changes include molecular modifications to DNA or chromatin and non-coding RNA expression. Among these, the most extensively investigated is DNA methylation, which takes place at the carbon-5 position of cytosine in CpG dinucleotides. DNMT1 is generally regarded as the maintenance methyltransferase to maintain DNA methylation following cell division and replication, while DNMT3A and DNMT3B are commonly regarded as *de novo* enzymes, essential for introducing new DNA methylation during early development [16]. It has been shown that cooperation of DNMT1 with DNMT3B is essential for efficient CpG island methylation of certain genes [17]. In the maternal low protein diet rat model of programming, the methylation pattern of the proximal promoter of the AT1b gene is changed significantly in the adrenal gland [5]. Goyal et al. demonstrated hypomethylation of the CpG islands in the promoter region of the ACE-1 gene in the fetal brain from the antenatal maternal low protein diet rat model [18]. Otherwise, 11β-hydroxysteroid dehydrogenase-2 (11βHSD-2) exhibits cell-specific expression in mineralocorticoid target tissues such as epithelial cells from renal cortical collecting tubules. Reduced activity of 11βHSD2 leads to overactivation of the mineralocorticoid receptor by cortisol with renal sodium retention, hypokalemia, and a salt-sensitive increase in blood pressure [19]. Alikhani-Koopaei et al. [20] found that proximal kidney tubules with hypermethylation of the HSD11B2 promoter almost do not express 11βHSD2 and DNA methyltransferase inhibitors decrease methylation of the HSD11B2 promoter of the kidney to upregulate its expression in cell lines and *in vivo*, indicating DNA methylation affecting HSD11B2 gene expression correlates with hypertension. Therefore, change of DNA methylation in the renal cortex tissue may be associated with developmental programming of hypertension and the expression and activity of DNMTs are very important for normal embryonic development [21].

Our previous studies found that prenatal exposure to LPS led to hypertension and increased body weight in adult offspring rats, and PDTC treatment could observably reversed the anomalies in blood pressure and body weight [2, 22]. In the current study, we successfully duplicated the rat model and found that expression of DNMT1 and DNMT3B in the renal cortex were increased significantly at both the mRNA and protein levels in the LPS group compared to the control. Meanwhile, the global DNA methylation level of renal cortex was increased dramatically in rat offspring of the LPS group. These results suggest that prenatal inflammatory exposure leads to increased DNMTs activity in the renal tissue of offspring.

Prenatal inflammatory exposure leads to increased inflammatory cytokines in the mother, fetus, and adult offspring [6]. Inflammatory cytokines change the expression and activity of DNMTs. IL-6 increases the expression of DNMT1 in colon cancer cells and enhances nuclear translocation of DNMT1 [7, 8, 23, 24]. IL-6 enhances the expression of DNMT1 and decreases the expression of lysyloxidase through the Fli-1 pathway [9, 25]. TNF-α stimulates DNMT3B expression, leading to histone methylations, silencing Notch-1 gene expression through NF-κB [10]. The current study shows upregulation of IL-6 and TNF-α expression of the renal cortex in prenatal inflammation-stimulated offspring, suggesting that increased inflammatory cytokines may contribute to renal DNMTs activity in adult offspring.

The transcription factor Fli-1, is essential for IL-6-dependent Dnmt1 stimulation [9]. In this study, we established that the mRNA and protein expression of Fli-1 in the renal cortex was increased significantly in the LPS group compared to the control. Together with our finding of increased DNMT1 and DNMT3B levels in the renal cortex of prenatal inflammation-stimulated offspring, these data suggest that IL-6-dependent Fli-1 elevation might play a critical role in the increased renal DNMTs activities in the offspring of prenatal inflammatory exposure.

As we know, LPS treatment can result in upregulation of NF-κB-dependent IL-6 and TNF-α expression [6, 7]. PDTC is a specific IκBα degradation inhibitor that selectively prevents NF-κB activation to inhibit NF-κB activity in both the mother and fetus in the model of prenatal inflammatory exposure [26]. In this study, the increased expression of IL-6, Fli-1, TNF-α, DNMT1 and DNMT3B, together with the developmental hypertension of prenatal inflammation-induced offspring were reversed by prenatal PDTC administration. These findings indicated that the increased inflammatory response caused further DNA methylation of specific genes during the development of adult hypertension.

In conclusion, prenatal exposure to inflammation results in higher NF-κB-dependent IL-6 and TNF-α expression of the renal cortex, leading to increased Fli-1 expression. The activation of IL-6/Fli-1 pathway and TNF-α might maintain the higher level of DNMTs and affects the methylation of certain key genes. These combined effects may silence or alter the expression of the key genes, leading to hypertension in adult offspring. Given the totality of our data, our future direction is to identify these key genes, which warrants further laboratory evaluation and clinical study.
